# Amber-red color imaging for enhanced visualization and hemostasis during rectal endoscopic submucosal dissection

**DOI:** 10.1055/a-2714-3208

**Published:** 2025-10-10

**Authors:** Chih-Wen Huang, Yang-Yuan Chen, Hsu-Heng Yen

**Affiliations:** 1 36596 Division of Gastroenterology, Changhua Christian Hospital, Changhua City, Taiwan

A 64-year-old man underwent colonoscopy due to a positive fecal occult blood test, which revealed an incidental 4-cm laterally spreading tumor (LST), mixed nodular type, in the rectum ( Fig. 1 ) Endoscopic submucosal dissection (ESD) was performed using a colonoscope (Fujifilm EC-760S-V/L) and the ELUXEO 8000 system (FUJIFILM Co., Tokyo, Japan), with the novel amber-red color imaging (ACI) mode applied throughout the procedure. Submucosal injection of indigo carmine mixed with glycerol provided lesion lifting. Under ACI mode, the blue contrast of the dye appeared more vivid, which enhanced visualization of fine vascular structures and clearly delineating the submucosal layer ( Fig. 2 , Fig. 3 , Video 1 ) Moreover, during intraoperative bleeding, ACI mode allowed precise identification of the bleeding point, aiding efficient hemostasis ( Fig. 4 , Video 1 ). Final histopathology confirmed a tubulovillous adenoma.

**Fig. 1:**
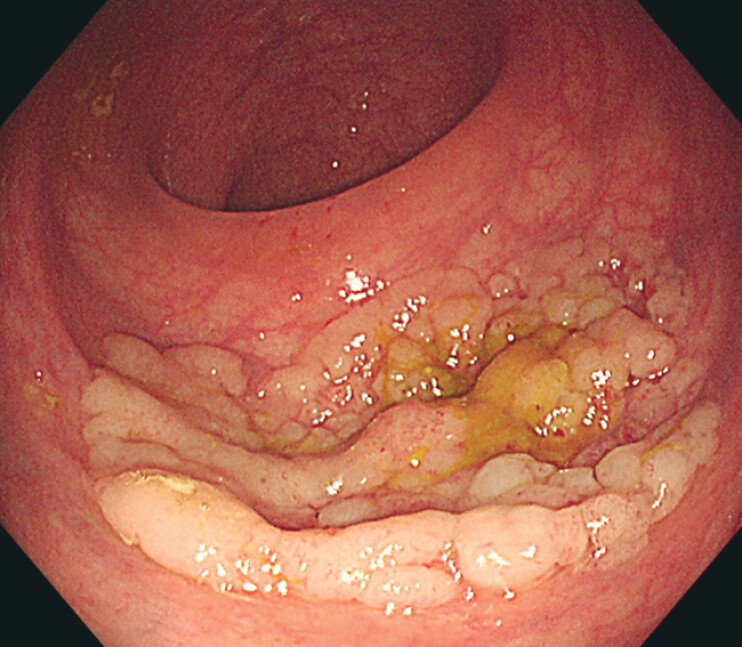
A 4-cm laterally spreading tumor (LST), mixed nodular type, in the rectum.

**Fig. 2:**
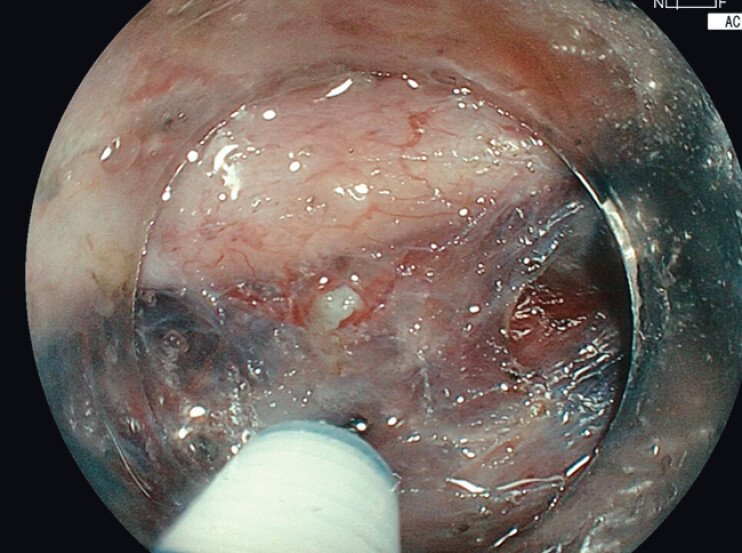
ACI mode enables clear visualization of fine vascular structures within the submucosa.

**Fig. 3:**
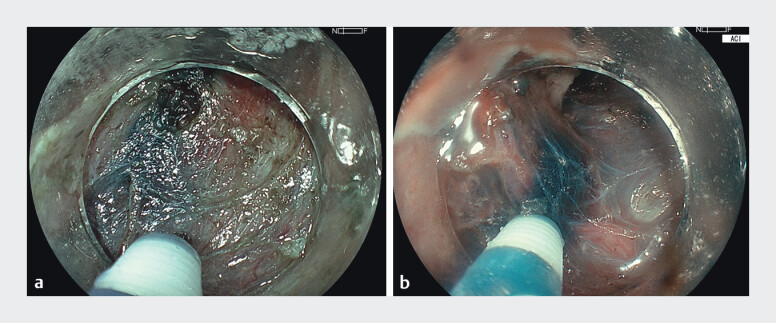
ACI mode preserves a color tone similar to white light imaging (WLI) while enhancing contrast—highlighting the blue-stained submucosa and orange-red vasculature—allowing it to serve as a more efficient imaging mode throughout ESD. **a** WLI mode. **b** ACI mode.

**Fig. 4:**
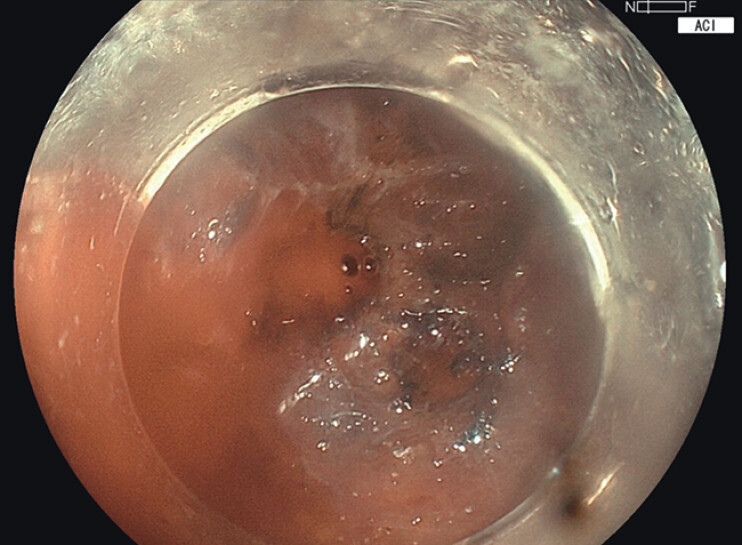
ACI mode highlights the bleeding point with an orange-red hue, enabling clear distinction from the surrounding blood pool. The enhanced color contrast facilitates rapid identification and targeted hemostasis.

This video demonstrates rectal ESD using amber-red color imaging (ACI). ACI enhanced visualization of tissue planes and fine vessels, improved bleeding point detection, and enabled precise hemostasis with a coagrasper. The procedure was completed safely and efficiently using ACI as a single imaging mode.Video 1

ACI mode utilizes amber-red, green, and blue light-emitting diodes (LEDs) to enhance spectral contrast and highlight subtle differences in blood coloration 1 . Like red dichromatic imaging (RDI), ACI enhances color contrast between the bleeder and the surrounding blood pool, facilitating accurate identification of bleeding sites 2 . However, ACI offers a more natural color tone, closely resembling white light imaging (WLI), making it more suitable for continuous use throughout ESD without the need to switch imaging modes, unlike RDI. By combining linked color imaging (LCI) with brightness enhancement, ACI intensifies red hues while preserving overall image familiarity, thus improving visualization of vascular structures, the submucosal plane, and the muscle layer—ultimately enhancing both safety and efficiency of ESD 3 .

## References

[1] Funasaka K Horiguchi N Yamada H Endoscopic submucosal dissection of early gastric cancer using a novel image-enhanced endoscopy: amber-red color imaging Endoscopy 2024 56 E640 e1 10.1055/a-2357-8351 39059452 PMC11281843

[2] Huang CW Yen HH Chen YY Successful hemostasis with red dichromatic imaging for bleeding rectal dieulafoy's lesion Endoscopy 2024 56 E160 e1 10.1055/a-2253-8797 38359890 PMC10869226

[3] Kanzaki H Takenaka R Kawahara Y Linked color imaging (LCI), a novel image-enhanced endoscopy technology, emphasizes the color of early gastric cancer Endosc Int Open 2017 5 E1005 e13 10.1055/s-0043-117881 29159276 PMC5634856

